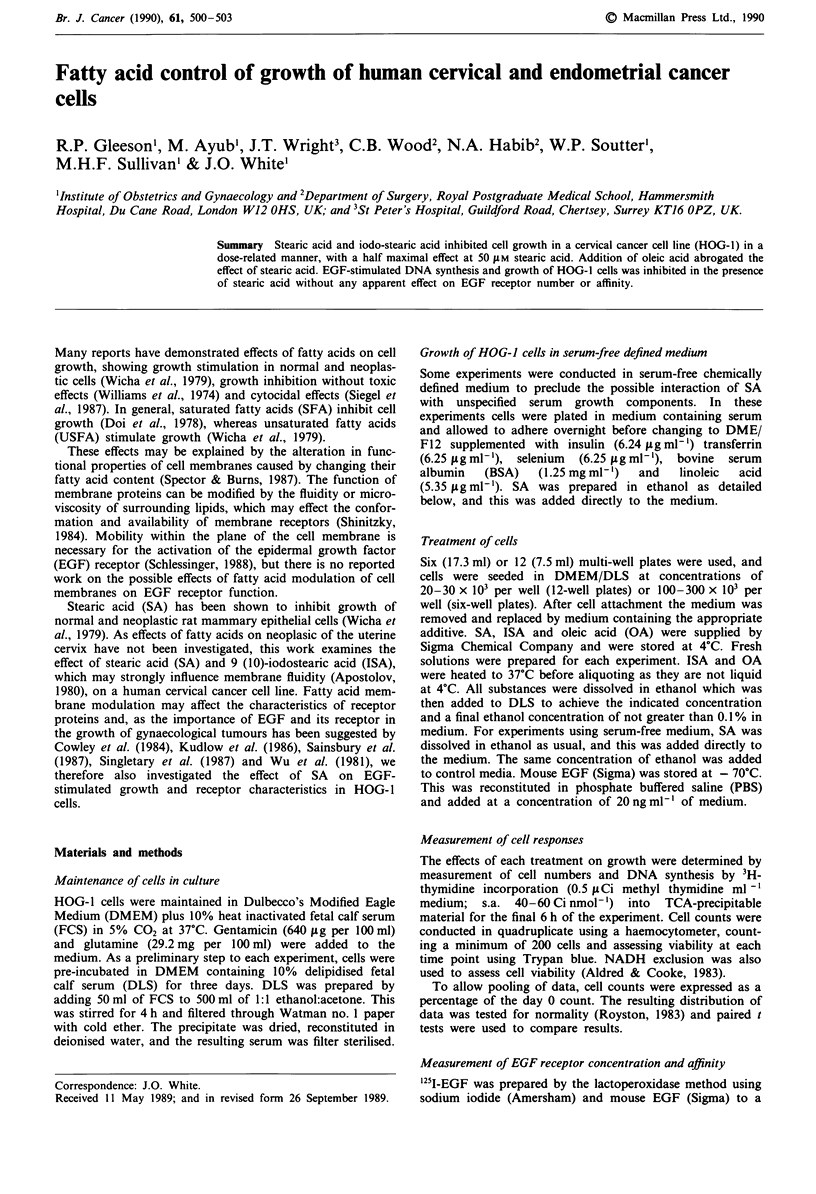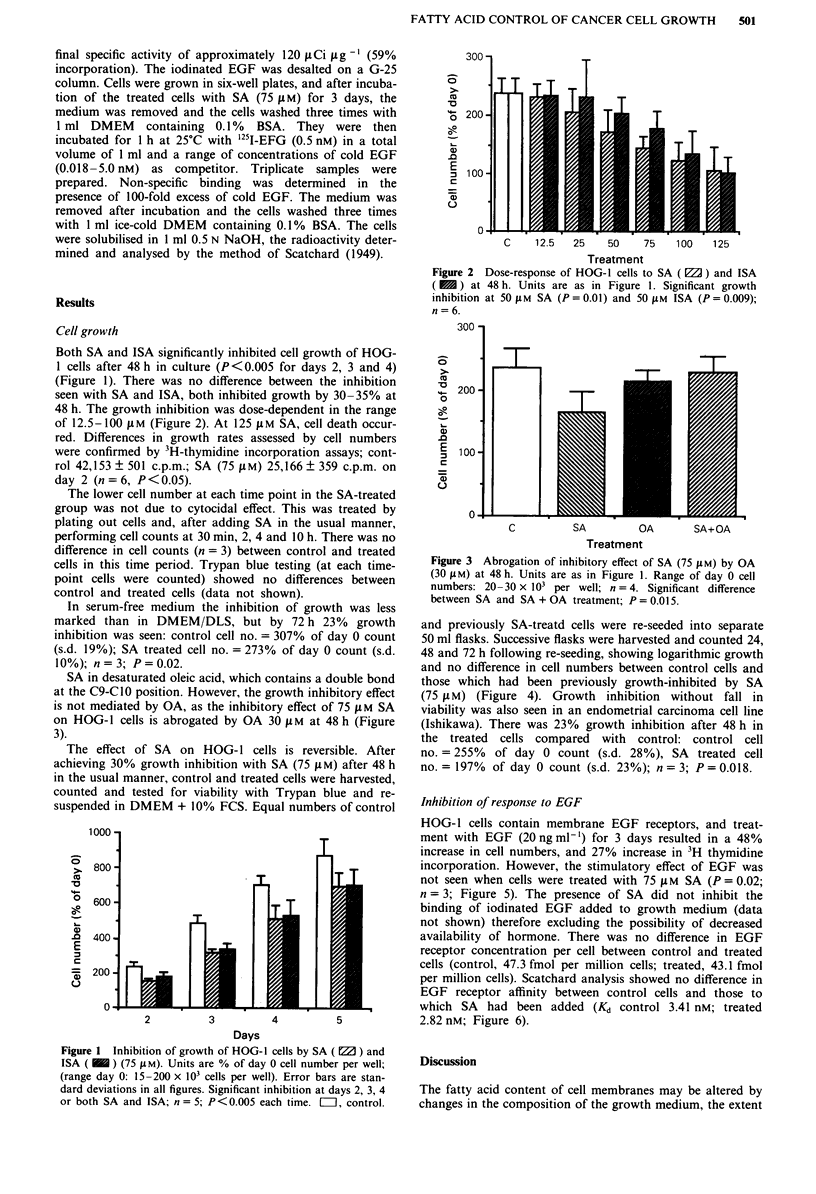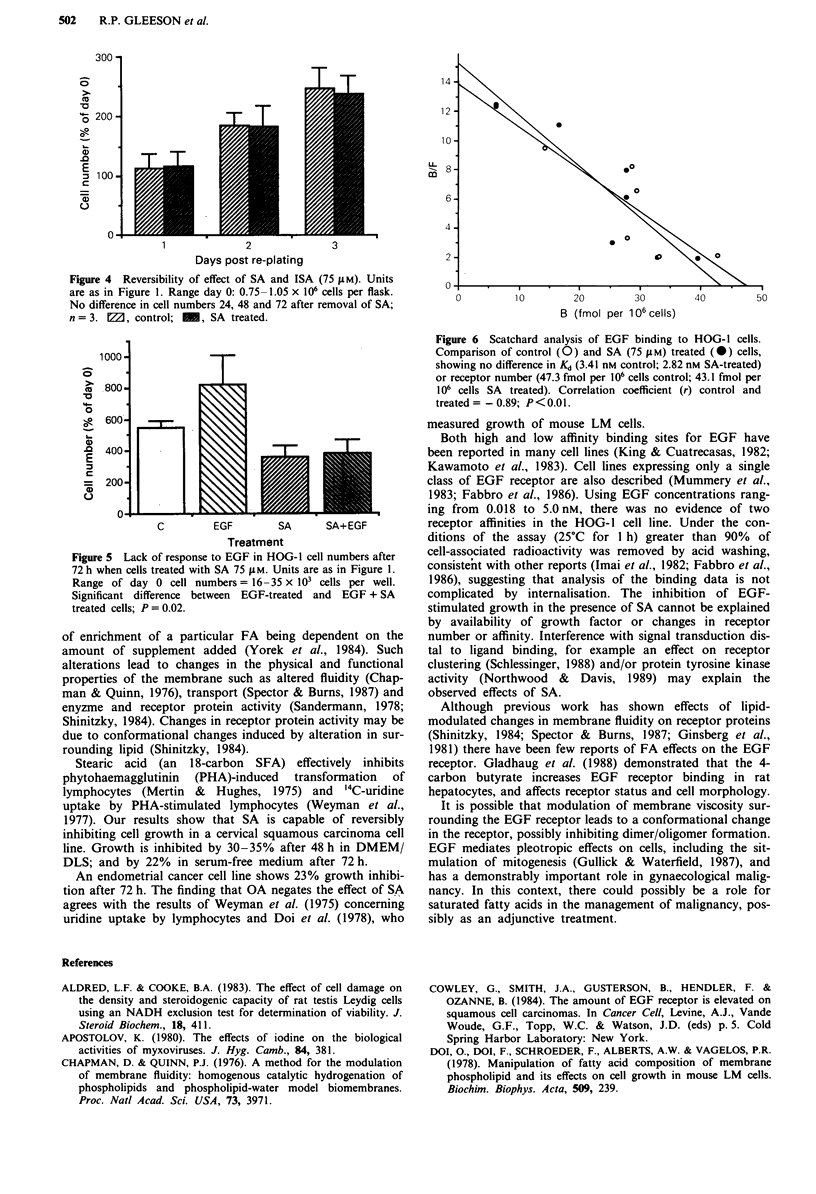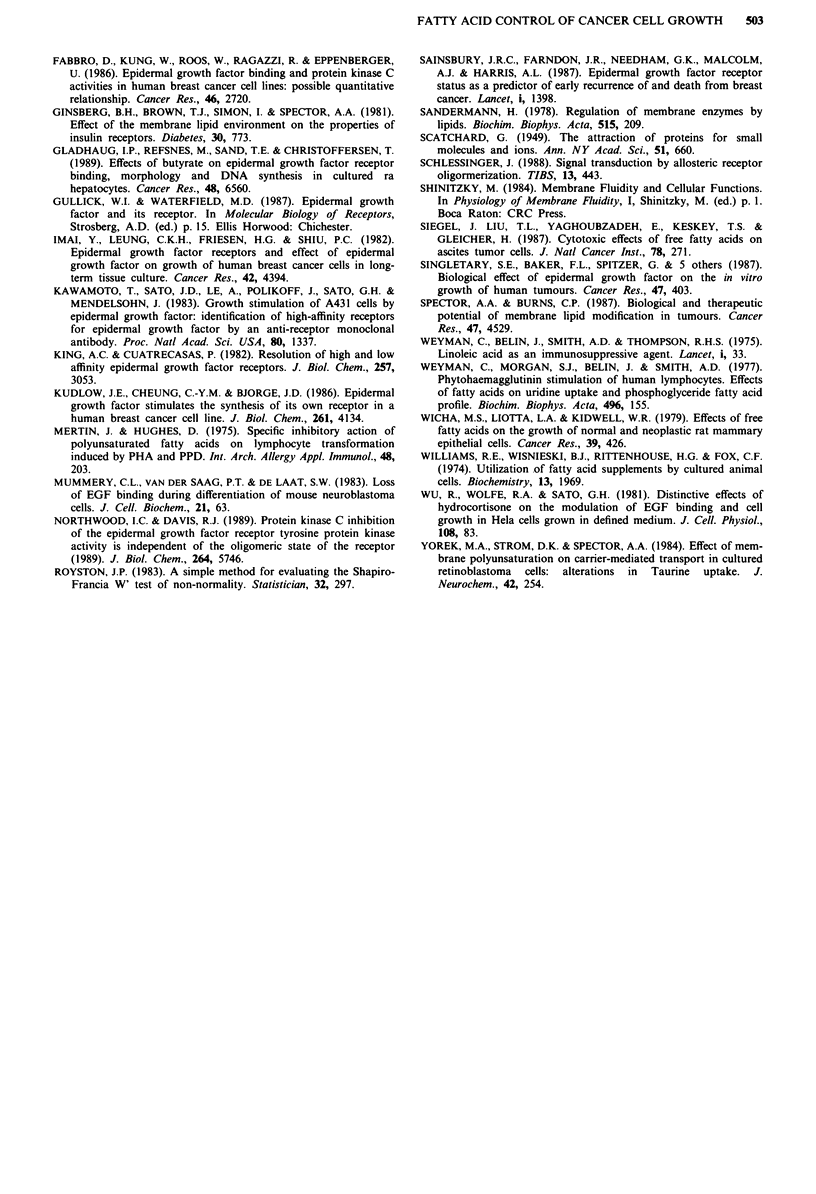# Fatty acid control of growth of human cervical and endometrial cancer cells.

**DOI:** 10.1038/bjc.1990.113

**Published:** 1990-04

**Authors:** R. P. Gleeson, M. Ayub, J. T. Wright, C. B. Wood, N. A. Habib, W. P. Soutter, M. H. Sullivan, J. O. White

**Affiliations:** Institute of Obstetrics and Gynaecology, Hammersmith Hospital, London, UK.

## Abstract

Stearic acid and iodo-stearic and inhibited cell growth in a cervical cancer cell line (HOG-1) in a dose-related manner, with a half maximal effect at 50 microM stearic acid. Addition of oleic acid abrogated the effect of stearic acid. EGF-stimulated DNA synthesis and growth of HOG-1 cells was inhibited in the presence of stearic acid without any apparent effect on EGF receptor number or affinity.


Br. J. Cancer (1990), 61, 500-503 © Macmillan Press Ltd., 1990

### Fatty acid control of growth of human cervical and endometrial cancer cells

R.P. Gleeson[1], M. Ayub[1], J.T. Wright[3], C.B. Wood[2], N.A. Habib[2], W.P. Soutter[1], M.H.F. Sullivan[1] & J.O. White[1]

[1]*Institute of Obstetrics and Gynaecology and* [2]*Department of Surgery, Royal Postgraduate Medical School, Hammersmith Hospital, Du Cane Road, London W12 0HS, UK; and* [3]*St Peter's Hospital, Guildford Road, Chertsey, Surrey KT16 0PZ, UK.*

**Summary** Stearic acid and iodo-stearic acid inhibited cell growth in a cervical cancer cell line (HOG-1) in a dose-related manner, with a half maximal effect at 50 μM stearic acid. Addition of oleic acid abrogated the effect of stearic acid. EGF-stimulated DNA synthesis and growth of HOG-1 cells was inhibited in the presence of stearic acid without any apparent effect on EGF receptor number or affinity.

Many reports have demonstrated effects of fatty acids on cell growth, showing growth stimulation in normal and neoplastic cells (Wicha *et al.*, 1979), growth inhibition without toxic effects (Williams *et al.*, 1974) and cytocidal effects (Siegel *et al.*, 1987). In general, saturated fatty acids (SFA) inhibit cell growth (Doi *et al.*, 1978), whereas unsaturated fatty acids (USFA) stimulate growth (Wicha *et al.*, 1979).

These effects may be explained by the alteration in functional properties of cell membranes caused by changing their fatty acid content (Spector & Burns, 1987). The function of membrane proteins can be modified by the fluidity or microviscosity of surrounding lipids, which may effect the conformation and availability of membrane receptors (Shinitzky, 1984). Mobility within the plane of the cell membrane is necessary for the activation of the epidermal growth factor (EGF) receptor (Schlessinger, 1988), but there is no reported work on the possible effects of fatty acid modulation of cell membranes on EGF receptor function.

Stearic acid (SA) has been shown to inhibit growth of normal and neoplastic rat mammary epithelial cells (Wicha *et al.*, 1979). As effects of fatty acids on neoplasic of the uterine cervix have not been investigated, this work examines the effect of stearic acid (SA) and 9 (10)-iodostearic acid (ISA), which may strongly influence membrane fluidity (Apostolov, 1980), on a human cervical cancer cell line. Fatty acid membrane modulation may affect the characteristics of receptor proteins and, as the importance of EGF and its receptor in the growth of gynaecological tumours has been suggested by Cowley *et al.* (1984), Kudlow *et al.* (1986), Sainsbury *et al.* (1987), Singletary *et al.* (1987) and Wu *et al.* (1981), we therefore also investigated the effect of SA on EGF-stimulated growth and receptor characteristics in HOG-1 cells.

### Materials and methods

### *Maintenance of cells in culture*

HOG-1 cells were maintained in Dulbecco's Modified Eagle Medium (DMEM) plus 10% heat inactivated fetal calf serum (FCS) in 5% $CO_2$ at 37°C. Gentamicin (640 μg per 100 ml) and glutamine (29.2 mg per 100 ml) were added to the medium. As a preliminary step to each experiment, cells were pre-incubated in DMEM containing 10% delipidised fetal calf serum (DLS) for three days. DLS was prepared by adding 50 ml of FCS to 500 ml of 1:1 ethanol:acetone. This was stirred for 4 h and filtered through Watman no. 1 paper with cold ether. The precipitate was dried, reconstituted in deionised water, and the resulting serum was filter sterilised.

Correspondence: J.O. White.
Received 11 May 1989; and in revised form 26 September 1989.

### *Growth of HOG-1 cells in serum-free defined medium*

Some experiments were conducted in serum-free chemically defined medium to preclude the possible interaction of SA with unspecified serum growth components. In these experiments cells were plated in medium containing serum and allowed to adhere overnight before changing to DME/F12 supplemented with insulin (6.24 $\mu g\,ml^{-1}$) transferrin (6.25 $\mu g\,ml^{-1}$), selenium (6.25 $\mu g\,ml^{-1}$), bovine serum albumin (BSA) (1.25 $mg\,ml^{-1}$) and linoleic acid (5.35 $\mu g\,ml^{-1}$). SA was prepared in ethanol as detailed below, and this was added directly to the medium.

### *Treatment of cells*

Six (17.3 ml) or 12 (7.5 ml) multi-well plates were used, and cells were seeded in DMEM/DLS at concentrations of $20-30 \times 10^3$ per well (12-well plates) or $100-300 \times 10^3$ per well (six-well plates). After cell attachment the medium was removed and replaced by medium containing the appropriate additive. SA, ISA and oleic acid (OA) were supplied by Sigma Chemical Company and were stored at 4°C. Fresh solutions were prepared for each experiment. ISA and OA were heated to 37°C before aliquoting as they are not liquid at 4°C. All substances were dissolved in ethanol which was then added to DLS to achieve the indicated concentration and a final ethanol concentration of not greater than 0.1% in medium. For experiments using serum-free medium, SA was dissolved in ethanol as usual, and this was added directly to the medium. The same concentration of ethanol was added to control media. Mouse EGF (Sigma) was stored at − 70°C. This was reconstituted in phosphate buffered saline (PBS) and added at a concentration of 20 $ng\,ml^{-1}$ of medium.

### *Measurement of cell responses*

The effects of each treatment on growth were determined by measurement of cell numbers and DNA synthesis by $^3H$-thymidine incorporation (0.5 μCi methyl thymidine $ml^{-1}$ medium; s.a. 40-60 Ci $nmol^{-1}$) into TCA-precipitable material for the final 6 h of the experiment. Cell counts were conducted in quadruplicate using a haemocytometer, counting a minimum of 200 cells and assessing viability at each time point using Trypan blue. NADH exclusion was also used to assess cell viability (Aldred & Cooke, 1983).

To allow pooling of data, cell counts were expressed as a percentage of the day 0 count. The resulting distribution of data was tested for normality (Royston, 1983) and paired *t* tests were used to compare results.

### *Measurement of EGF receptor concentration and affinity*

$^{125}I$-EGF was prepared by the lactoperoxidase method using sodium iodide (Amersham) and mouse EGF (Sigma) to a final specific activity of approximately 120 μCi μg$^{-1}$ (59% incorporation). The iodinated EGF was desalted on a G-25 column. Cells were grown in six-well plates, and after incubation of the treated cells with SA (75 μM) for 3 days, the medium was removed and the cells washed three times with 1 ml DMEM containing 0.1% BSA. They were then incubated for 1 h at 25°C with $^{125}$I-EFG (0.5 nM) in a total volume of 1 ml and a range of concentrations of cold EGF (0.018–5.0 nM) as competitor. Triplicate samples were prepared. Non-specific binding was determined in the presence of 100-fold excess of cold EGF. The medium was removed after incubation and the cells washed three times with 1 ml ice-cold DMEM containing 0.1% BSA. The cells were solubilised in 1 ml 0.5 N NaOH, the radioactivity determined and analysed by the method of Scatchard (1949).

### Results

### *Cell growth*

Both SA and ISA significantly inhibited cell growth of HOG-1 cells after 48 h in culture ($P<0.005$ for days 2, 3 and 4) (Figure 1). There was no difference between the inhibition seen with SA and ISA, both inhibited growth by 30–35% at 48 h. The growth inhibition was dose-dependent in the range of 12.5–100 μM (Figure 2). At 125 μM SA, cell death occurred. Differences in growth rates assessed by cell numbers were confirmed by $^{3}$H-thymidine incorporation assays; control 42,153 ± 501 c.p.m.; SA (75 μM) 25,166 ± 359 c.p.m. on day 2 ($n = 6$, $P<0.05$).

The lower cell number at each time point in the SA-treated group was not due to cytocidal effect. This was treated by plating out cells and, after adding SA in the usual manner, performing cell counts at 30 min, 2, 4 and 10 h. There was no difference in cell counts ($n = 3$) between control and treated cells in this time period. Trypan blue testing (at each time-point cells were counted) showed no differences between control and treated cells (data not shown).

In serum-free medium the inhibition of growth was less marked than in DMEM/DLS, but by 72 h 23% growth inhibition was seen: control cell no. = 307% of day 0 count (s.d. 19%); SA treated cell no. = 273% of day 0 count (s.d. 10%); $n = 3$; $P = 0.02$.

SA in desaturated oleic acid, which contains a double bond at the C9-C10 position. However, the growth inhibitory effect is not mediated by OA, as the inhibitory effect of 75 μM SA on HOG-1 cells is abrogated by OA 30 μM at 48 h (Figure 3).

The effect of SA on HOG-1 cells is reversible. After achieving 30% growth inhibition with SA (75 μM) after 48 h in the usual manner, control and treated cells were harvested, counted and tested for viability with Trypan blue and re-suspended in DMEM + 10% FCS. Equal numbers of control

**Figure 1** Inhibition of growth of HOG-1 cells by SA ( ) and ISA ( ) (75 μM). Units are % of day 0 cell number per well; (range day 0: 15–200 × 10$^{3}$ cells per well). Error bars are standard deviations in all figures. Significant inhibition at days 2, 3, 4 or both SA and ISA; $n = 5$; $P<0.005$ each time. ( ), control.

**Figure 2** Dose-response of HOG-1 cells to SA ( ) and ISA ( ) at 48 h. Units are as in Figure 1. Significant growth inhibition at 50 μM SA ($P = 0.01$) and 50 μM ISA ($P = 0.009$); $n = 6$.

**Figure 3** Abrogation of inhibitory effect of SA (75 μM) by OA (30 μM) at 48 h. Units are as in Figure 1. Range of day 0 cell numbers: 20–30 × 10$^{3}$ per well; $n = 4$. Significant difference between SA and SA + OA treatment; $P = 0.015$.

and previously SA-treatd cells were re-seeded into separate 50 ml flasks. Successive flasks were harvested and counted 24, 48 and 72 h following re-seeding, showing logarithmic growth and no difference in cell numbers between control cells and those which had been previously growth-inhibited by SA (75 μM) (Figure 4). Growth inhibition without fall in viability was also seen in an endometrial carcinoma cell line (Ishikawa). There was 23% growth inhibition after 48 h in the treated cells compared with control: control cell no. = 255% of day 0 count (s.d. 28%), SA treated cell no. = 197% of day 0 count (s.d. 23%); $n = 3$; $P = 0.018$.

### *Inhibition of response to EGF*

HOG-1 cells contain membrane EGF receptors, and treatment with EGF (20 ng ml$^{-1}$) for 3 days resulted in a 48% increase in cell numbers, and 27% increase in $^{3}$H thymidine incorporation. However, the stimulatory effect of EGF was not seen when cells were treated with 75 μM SA ($P = 0.02$; $n = 3$; Figure 5). The presence of SA did not inhibit the binding of iodinated EGF added to growth medium (data not shown) therefore excluding the possibility of decreased availability of hormone. There was no difference in EGF receptor concentration per cell between control and treated cells (control, 47.3 fmol per million cells; treated, 43.1 fmol per million cells). Scatchard analysis showed no difference in EGF receptor affinity between control cells and those to which SA had been added ($K_d$ control 3.41 nM; treated 2.82 nM; Figure 6).

### Discussion

The fatty acid content of cell membranes may be altered by changes in the composition of the growth medium, the extent

**Figure 4** Reversibility of effect of SA and ISA (75 µM). Units are as in Figure 1. Range day 0: 0.75–1.05 × $10^6$ cells per flask. No difference in cell numbers 24, 48 and 72 after removal of SA; $n = 3$. ▨, control; ■, SA treated.

**Figure 5** Lack of response to EGF in HOG-1 cell numbers after 72 h when cells treated with SA 75 µM. Units are as in Figure 1. Range of day 0 cell numbers = 16–35 × $10^3$ cells per well. Significant difference between EGF-treated and EGF + SA treated cells; $P = 0.02$.

of enrichment of a particular FA being dependent on the amount of supplement added (Yorek *et al.*, 1984). Such alterations lead to changes in the physical and functional properties of the membrane such as altered fluidity (Chapman & Quinn, 1976), transport (Spector & Burns, 1987) and enyzme and receptor protein activity (Sandermann, 1978; Shinitzky, 1984). Changes in receptor protein activity may be due to conformational changes induced by alteration in surrounding lipid (Shinitzky, 1984).

Stearic acid (an 18-carbon SFA) effectively inhibits phytohaemagglutinin (PHA)-induced transformation of lymphocytes (Mertin & Hughes, 1975) and $^{14}C$-uridine uptake by PHA-stimulated lymphocytes (Weyman *et al.*, 1977). Our results show that SA is capable of reversibly inhibiting cell growth in a cervical squamous carcinoma cell line. Growth is inhibited by 30–35% after 48 h in DMEM/DLS; and by 22% in serum-free medium after 72 h.

An endometrial cancer cell line shows 23% growth inhibition after 72 h. The finding that OA negates the effect of SA agrees with the results of Weyman *et al.* (1975) concerning uridine uptake by lymphocytes and Doi *et al.* (1978), who

**Figure 6** Scatchard analysis of EGF binding to HOG-1 cells. Comparison of control (○) and SA (75 µM) treated (●) cells, showing no difference in $K_d$ (3.41 nM control; 2.82 nM SA-treated) or receptor number (47.3 fmol per $10^6$ cells control; 43.1 fmol per $10^6$ cells SA treated). Correlation coefficient ($r$) control and treated = − 0.89; $P < 0.01$.

measured growth of mouse LM cells.

Both high and low affinity binding sites for EGF have been reported in many cell lines (King & Cuatrecasas, 1982; Kawamoto *et al.*, 1983). Cell lines expressing only a single class of EGF receptor are also described (Mummery *et al.*, 1983; Fabbro *et al.*, 1986). Using EGF concentrations ranging from 0.018 to 5.0 nM, there was no evidence of two receptor affinities in the HOG-1 cell line. Under the conditions of the assay (25°C for 1 h) greater than 90% of cell-associated radioactivity was removed by acid washing, consistent with other reports (Imai *et al.*, 1982; Fabbro *et al.*, 1986), suggesting that analysis of the binding data is not complicated by internalisation. The inhibition of EGF-stimulated growth in the presence of SA cannot be explained by availability of growth factor or changes in receptor number or affinity. Interference with signal transduction distal to ligand binding, for example an effect on receptor clustering (Schlessinger, 1988) and/or protein tyrosine kinase activity (Northwood & Davis, 1989) may explain the observed effects of SA.

Although previous work has shown effects of lipid-modulated changes in membrane fluidity on receptor proteins (Shinitzky, 1984; Spector & Burns, 1987; Ginsberg *et al.*, 1981) there have been few reports of FA effects on the EGF receptor. Gladhaug *et al.* (1988) demonstrated that the 4-carbon butyrate increases EGF receptor binding in rat hepatocytes, and affects receptor status and cell morphology.

It is possible that modulation of membrane viscosity surrounding the EGF receptor leads to a conformational change in the receptor, possibly inhibiting dimer/oligomer formation. EGF mediates pleotropic effects on cells, including the sitmulation of mitogenesis (Gullick & Waterfield, 1987), and has a demonstrably important role in gynaecological malignancy. In this context, there could possibly be a role for saturated fatty acids in the management of malignancy, possibly as an adjunctive treatment.

### References

ALDRED, L.F. & COOKE, B.A. (1983). The effect of cell damage on the density and steroidogenic capacity of rat testis Leydig cells using an NADH exclusion test for determination of viability. *J. Steroid Biochem.*, **18**, 411.

APOSTOLOV, K. (1980). The effects of iodine on the biological activities of myxoviruses. *J. Hyg. Camb.*, **84**, 381.

CHAPMAN, D. & QUINN, P.J. (1976). A method for the modulation of membrane fluidity: homogenous catalytic hydrogenation of phospholipids and phospholipid-water model biomembranes. *Proc. Natl Acad. Sci. USA*, **73**, 3971.

COWLEY, G., SMITH, J.A., GUSTERSON, B., HENDLER, F. & OZANNE, B. (1984). The amount of EGF receptor is elevated on squamous cell carcinomas. In *Cancer Cell*, Levine, A.J., Vande Woude, G.F., Topp, W.C. & Watson, J.D. (eds) p. 5. Cold Spring Harbor Laboratory: New York.

DOI, O., DOI, F., SCHROEDER, F., ALBERTS, A.W. & VAGELOS, P.R. (1978). Manipulation of fatty acid composition of membrane phospholipid and its effects on cell growth in mouse LM cells. *Biochim. Biophys. Acta*, **509**, 239.

FABBRO, D., KUNG, W., ROOS, W., RAGAZZI, R. & EPPENBERGER, U. (1986). Epidermal growth factor binding and protein kinase C activities in human breast cancer cell lines: possible quantitative relationship. *Cancer Res.*, **46,** 2720.

GINSBERG, B.H., BROWN, T.J., SIMON, I. & SPECTOR, A.A. (1981). Effect of the membrane lipid environment on the properties of insulin receptors. *Diabetes*, **30,** 773.

GLADHAUG, I.P., REFSNES, M., SAND, T.E. & CHRISTOFFERSEN, T. (1989). Effects of butyrate on epidermal growth factor receptor binding, morphology and DNA synthesis in cultured ra hepatocytes. *Cancer Res.*, **48,** 6560.

GULLICK, W.I. & WATERFIELD, M.D. (1987). Epidermal growth factor and its receptor. In *Molecular Biology of Receptors*, Strosberg, A.D. (ed.) p. 15. Ellis Horwood: Chichester.

IMAI, Y., LEUNG, C.K.H., FRIESEN, H.G. & SHIU, P.C. (1982). Epidermal growth factor receptors and effect of epidermal growth factor on growth of human breast cancer cells in long-term tissue culture. *Cancer Res.*, **42,** 4394.

KAWAMOTO, T., SATO, J.D., LE, A., POLIKOFF, J., SATO, G.H. & MENDELSOHN, J. (1983). Growth stimulation of A431 cells by epidermal growth factor: identification of high-affinity receptors for epidermal growth factor by an anti-receptor monoclonal antibody. *Proc. Natl Acad. Sci. USA*, **80,** 1337.

KING, A.C. & CUATRECASAS, P. (1982). Resolution of high and low affinity epidermal growth factor receptors. *J. Biol. Chem.*, **257,** 3053.

KUDLOW, J.E., CHEUNG, C.-Y.M. & BJORGE, J.D. (1986). Epidermal growth factor stimulates the synthesis of its own receptor in a human breast cancer cell line. *J. Biol. Chem.*, **261,** 4134.

MERTIN, J. & HUGHES, D. (1975). Specific inhibitory action of polyunsaturated fatty acids on lymphocyte transformation induced by PHA and PPD. *Int. Arch. Allergy Appl. Immunol.*, **48,** 203.

MUMMERY, C.L., VAN DER SAAG, P.T. & DE LAAT, S.W. (1983). Loss of EGF binding during differentiation of mouse neuroblastoma cells. *J. Cell. Biochem.*, **21,** 63.

NORTHWOOD, I.C. & DAVIS, R.J. (1989). Protein kinase C inhibition of the epidermal growth factor receptor tyrosine protein kinase activity is independent of the oligomeric state of the receptor (1989). *J. Biol. Chem.*, **264,** 5746.

ROYSTON, J.P. (1983). A simple method for evaluating the Shapiro-Francia W' test of non-normality. *Statistician*, **32,** 297.

SAINSBURY, J.R.C., FARNDON, J.R., NEEDHAM, G.K., MALCOLM, A.J. & HARRIS, A.L. (1987). Epidermal growth factor receptor status as a predictor of early recurrence of and death from breast cancer. *Lancet*, **i,** 1398.

SANDERMANN, H. (1978). Regulation of membrane enzymes by lipids. *Biochim. Biophys. Acta*, **515,** 209.

SCATCHARD, G. (1949). The attraction of proteins for small molecules and ions. *Ann. NY Acad. Sci.*, **51,** 660.

SCHLESSINGER, J. (1988). Signal transduction by allosteric receptor oligormerization. *TIBS*, **13,** 443.

SHINITZKY, M. (1984). Membrane Fluidity and Cellular Functions. In *Physiology of Membrane Fluidity*, I, Shinitzky, M. (ed.) p. 1. Boca Raton: CRC Press.

SIEGEL, J. LIU, T.L., YAGHOUBZADEH, E., KESKEY, T.S. & GLEICHER, H. (1987). Cytotoxic effects of free fatty acids on ascites tumor cells. *J. Natl Cancer Inst.*, **78,** 271.

SINGLETARY, S.E., BAKER, F.L., SPITZER, G. & 5 others (1987). Biological effect of epidermal growth factor on the *in vitro* growth of human tumours. *Cancer Res.*, **47,** 403.

SPECTOR, A.A. & BURNS, C.P. (1987). Biological and therapeutic potential of membrane lipid modification in tumours. *Cancer Res.*, **47,** 4529.

WEYMAN, C., BELIN, J., SMITH, A.D. & THOMPSON, R.H.S. (1975). Linoleic acid as an immunosuppressive agent. *Lancet*, **i,** 33.

WEYMAN, C., MORGAN, S.J., BELIN, J. & SMITH, A.D. (1977). Phytohaemagglutinin stimulation of human lymphocytes. Effects of fatty acids on uridine uptake and phosphoglyceride fatty acid profile. *Biochim. Biophys. Acta*, **496,** 155.

WICHA, M.S., LIOTTA, L.A. & KIDWELL, W.R. (1979). Effects of free fatty acids on the growth of normal and neoplastic rat mammary epithelial cells. *Cancer Res.*, **39,** 426.

WILLIAMS, R.E., WISNIESKI, B.J., RITTENHOUSE, H.G. & FOX, C.F. (1974). Utilization of fatty acid supplements by cultured animal cells. *Biochemistry*, **13,** 1969.

WU, R., WOLFE, R.A. & SATO, G.H. (1981). Distinctive effects of hydrocortisone on the modulation of EGF binding and cell growth in Hela cells grown in defined medium. *J. Cell. Physiol.*, **108,** 83.

YOREK, M.A., STROM, D.K. & SPECTOR, A.A. (1984). Effect of membrane polyunsaturation on carrier-mediated transport in cultured retinoblastoma cells: alterations in Taurine uptake. *J. Neurochem.*, **42,** 254.